# MRI after Interventional Therapy of Hepatocellular Carcinoma: Typical Changes over Time

**DOI:** 10.1055/a-2724-6488

**Published:** 2025-11-21

**Authors:** Jens Kübler, Antonia Ashkar, Moritz T. Winkelmann, Cihan Gani, Konstantin Nikolaou, Rüdiger Hoffmann

**Affiliations:** 1 27203 Department of Diagnostic and Interventional Radiology, Universitätsklinikum Tübingen, Tuebingen, Germany; 2 27203 Department of Radiation Oncology, Universitätsklinikum Tübingen, Tuebingen, Germany

**Keywords:** hepatocellular carcinoma, interventional procedures, MR-imaging, abdomen

## Abstract

**Background:**

Interventional therapies for hepatocellular carcinoma (HCC) include various methods such as transarterial chemoembolization (TACE), radiofrequency and microwave ablation (RFA, MWA), selective internal radiotherapy (SIRT), and stereotactic body radiation therapy (SBRT). Imaging follow-up using magnetic resonance imaging (MRI) is essential for the early detection of recurrence, but requires profound knowledge of therapy-related imaging changes.

**Materials & Methods:**

A structured PubMed literature search covering the period from 2000 to 2025 was conducted using the keywords “hepatocellular carcinoma,” “magnetic resonance imaging,” “thermal ablation,” “transarterial chemoembolization,” “transarterial radioembolization,” “stereotactic body radiotherapy,” and “treatment response.” In addition, the study incorporated current national and international guidelines, as well as institutional clinical experience.

**Results:**

Post-interventional imaging changes on MRI vary depending on the therapeutic approach applied. Typical morphological changes are observed immediately after thermoablation and TACE, whereas therapeutic effects of SIRT and SBRT become clearly evident only after weeks to months. Profound knowledge of standardized evaluation systems such as mRECIST, EASL, and LI-RADS-TR is crucial to ensure a precise and structured assessment of therapeutic response.

**Conclusion:**

Knowledge of specific MRI findings and standardized assessment systems is essential for structured follow-up of hepatocellular carcinoma after interventional therapy. Limitations arise from the heterogeneity of imaging findings, variable temporal patterns, and potential influences of combination therapies. Systemic therapies were not considered, restricting generalizability.

**Key Points:**

- Magnetic resonance imaging is essential for assessing treatment response and for the early detection of recurrence after interventional therapy of hepatocellular carcinoma.
- Morphological changes after therapy are procedure-dependent and show marked differences over time: SIRT and SBRT demonstrate delayed changes, whereas TACE and thermal ablation present immediate effects.
- Standardized classification systems (mRECIST, EASL, LI-RADS-TR) provide a structured and reproducible framework for evaluating treatment response.
- Knowledge of characteristic imaging findings and potential pitfalls is crucial to reliably differentiate between residual tumor, recurrence, and therapy-induced changes.
- This review contributes to the current body of knowledge by systematically consolidating available evidence and presenting it in a practice-oriented manner in the context of MRI follow-up imaging.

**Citation Format:**

- Kübler J, Ashkar A, Winkelmann MT et al. MRI after Interventional Therapy of Hepatocellular Carcinoma: Typical Changes over Time. Rofo 2025; DOI 10.1055/a-2724-6488

## Abbreviations

ADC: Apparent Diffusion Coefficient
AFP: Alpha-fetoprotein
BCLC: Barcelona Clinic Liver Cancer
CR: Complete response
CT: Computed tomography
cTACE: Conventional transarterial chemoembolization
DEB-TACE: Drug-eluting bead transarterial chemoembolization
DWI: Diffusion-weighted imaging
EASL: European Association for the Study of the Liver
FLR: Focal liver reaction
HCC: Hepatocellular carcinoma
INR: International normalized ratio
LI-RADS-TR: Liver Imaging Reporting and Data System – Treatment Response
mRECIST: Modified Response Evaluation Criteria in Solid Tumors
MRI: Magnetic resonance imaging
MWA: Microwave ablation
PD: Progressive disease
PR: Partial response
RFA: Radiofrequency ablation
RS: Radiation segmentectomy
RL: Radiation lobectomy
SBRT: Stereotactic body radiotherapy
SD: Stable disease
SIRT: Selective internal radiotherapy
TACE: Transarterial chemoembolization
TRA: Treatment response assessment
US: Ultrasound
VIBE: Volumetric interpolated breath-hold examination

## Introduction

Over the past two decades, interventional radiology has become integral to the multimodal treatment of hepatocellular carcinoma (HCC). Particularly for inoperable tumors, important treatment options include local therapies, such as thermal ablation using radiofrequency ablation (RFA) or microwave ablation (MWA), transarterial chemoembolization (TACE), and selective internal radiotherapy (SIRT) 1 . In addition, there is increasing use of high-precision percutaneous radiotherapy (stereotactic body radiotherapy, SBRT) 2 .

Imaging plays a central role in all stages of interventional therapy: from initial treatment planning to post-procedural success monitoring, and the exclusion of residual tumor, as well as in structured aftercare to detect any progression at an early stage. Among imaging techniques, magnetic resonance imaging (MRI) is considered the method of choice because it offers higher sensitivity with equivalent specificity compared to computed tomography, especially for lesions less than 2 cm in size. Due to its superior soft tissue contrast, it provides more precise lesion characterization and is thus particularly suitable for post-interventional follow-up 3 . In this context, it takes detailed knowledge of treatment-specific image patterns to assess the morphological response and detect therapy-induced changes 4 5 6 7 .

Standardized classification systems, such as mRECIST, EASL, and the treatment response module of the LI-RADS classification, provide structured criteria for assessing radiological treatment response. Each of the classification systems has different strengths, and it is also important to understand their respective limitations 5 8 9 .

The aim of this review article is to present the key aspects of post-interventional magnetic resonance imaging following local treatment procedures for hepatocellular carcinoma.

## Materials & Methods

For this review, a structured literature search was conducted in PubMed, covering the period from January 2000 to March 2025. The search terms used were “hepatocellular carcinoma,” “magnetic resonance imaging,” “thermal ablation,” “transarterial chemoembolization,” “transarterial radioembolization,” “stereotactic body radiotherapy,” and “treatment response.” We included English and German-language original articles and reviews about post-interventional imaging for HCC. Case reports were excluded. A total of 1,689 publications were identified. After removing duplicates and excluding thematically inappropriate papers, 281 articles remained. After screening the abstracts, 193 papers were shortlisted. Of these, we reviewed 98 full text versions. In addition, current national and international guidelines were taken into account, as well as in-house clinical experience.

## Interventional treatment methods

### Transarterial chemoembolization (TACE, DEB-TACE)

TACE is the recommended standard therapy for patients with intermediate-stage HCC (BCLC B), if curative options such as resection, transplantation, or ablation are not possible. The prerequisite is preserved liver function (Child-Pugh A to B7) and the absence of extrahepatic metastases or macroscopic vascular invasion 3 . The procedure is based on the predominantly arterial supply of the HCC, while the adjacent liver tissue is supplied by portal veins. TACE is performed on a selective basis and can be used as conventional TACE (cTACE), where a chemotherapeutic agent is administered intraarterially with Lipiodol as the carrier medium. Alternatively, with DEB-TACE (drug-eluting bead transarterial chemoembolization) drug-loaded embolization particles are used. The aim is not only to prevent ischemia-induced tumor necrosis but also to achieve a local cytostatic effect with low systemic toxicity. The embolic effect is present immediately after therapy and can be detected in imaging as a lack of arterial hyper-enhancement.

### Thermal ablations (RFA, MWA)

Thermal ablation using radiofrequency ablation (RFA) or microwave ablation (MWA) represents a curative treatment option for patients with early hepatocellular carcinoma (BCLC 0 and A), particularly for tumors with a diameter of up to three centimeters and a limited number of lesions. The prerequisite is preserved liver function and the absence of macroscopic vascular invasion or extrahepatic metastasis 3 . Radiofrequency ablation (RFA) and microwave ablation (MWA) differ in their technical approach, but both aim to induce irreversible coagulation necrosis of the tumor through locally induced hyperthermia. While RFA is based on an alternating current in the range of 400–500 kHz, MWA uses electromagnetic waves with frequencies in the range of 915 MHz to 2.45 GHz. Compared to RFA, MWA offers higher energy penetration, shorter application times, and less susceptibility to the “heat sink effect,” which refers to unwanted heat dissipation through large vessels near the target lesion 10 11 . The thermally induced necrosis zone can be detected in the MRI scan immediately after therapy, and it presents with a regression in size over the longer term.

### Selective internal radiotherapy (SIRT)

Selective internal radiotherapy (SIRT), also called transarterial radioembolization (TARE), is used in patients with advanced or intermediate-stage HCC, especially when other locoregional procedures are unsuitable or have been exhausted 3 12 . β-Emitting microspheres, usually yttrium-90, are administered selectively via the tumor arterial supply. Due to the radiation’s low penetration depth of 2.4 mm on average, selective treatment can be carried out and the surrounding tissue can be protected 13 . Pre-therapeutically, angiographic simulation with technetium-99m MAA is performed to exclude pulmonary or gastrointestinal shunts so that no unwanted extrahepatic accumulation occurs during therapy. Indications exist for portal venous tumor invasion without decompensated liver cirrhosis, as well as for inoperable tumors with preserved liver function (Child-Pugh A to B7). Originally used as a palliative measure in advanced disease, TARE is now increasingly being used for bridging therapy before liver transplantation, for downstaging therapy, as curative therapy in the context of radiation segmentectomy (RS), and for radiation lobectomy (RL) in preparation for resection 14 . The embolic effect is in the background compared to radiation damage, so that post-therapeutic changes in imaging only become apparent in the longer term 15 .

### Stereotactic radiotherapy (SBRT)

According to the current S3 guideline, stereotactic radiotherapy (SBRT) is a non-invasive treatment option for patients when thermal ablation or resection is not technically feasible or contraindicated or in the event of recurrence after thermal ablation 3 . It can be used in different stages of HCC 3 16 17 . In addition, SBRT is also increasingly used as a bridging or downstaging strategy before liver transplantation. SBRT enables targeted tumor necrosis through precise dose application with the best possible protection of the surrounding parenchyma 18 . The pathophysiological effects of SBRT are based on both direct DNA double-strand breaks in tumor cells and secondary biological processes, such as endothelial damage and a modulation of the tumor-associated microenvironment, which can promote immune-mediated antitumoral effects 19 . Because these processes are delayed, changes that can be detected using image morphology only occur over time 20 .

## Classification systems for treatment evaluation

The evaluation of treatment response after locoregional procedures in hepatocellular carcinoma requires specific classification systems, as conventional size measurement according to Response Evaluation Criteria in Solid Tumors (RECIST) only inadequately reflects treatment success or failure. The evaluation of contrast agent behavior, especially arterial hypervascularization and washout phenomenon, is crucial for assessing tumor viability. For standardized reporting, various systems have been developed, such as modified RECIST (mRECIST), the European Association for the Study of the Liver (EASL) criteria, and the Liver Imaging Reporting and Data System Treatment Response (LI-RADS-TR) algorithm, each of which has different focuses and limitations. With version 2024, the LI-RADS Treatment Response Assessment (TRA) has been fundamentally overhauled. For the first time, a distinction is made between a non-radiation TRA core for ablation and embolization therapies and a radiation TRA core for radiation-based procedures (SIRT, SBRT) 21 22 23 . A structured application of these systems is crucial for valid treatment monitoring and continued clinical decision-making 5 8 24 .

### RECIST 1.1

The Response Evaluation Criteria in Solid Tumors in version 1.1 define treatment response exclusively by size changes of measurable lesions (largest longitudinal diameter). A complete response (CR) corresponds to the disappearance of all lesions, a partial response (PR) requires at least a 30% diameter reduction, while progression (PD) is interpreted as an increase ≥20% or the appearance of new lesions. A stable finding is present if the change in size does not correspond to CR, PR, or PD 25 .

However, after locoregional therapy of HCC, avascular (but morphologically persistent) lesions often remain, so that RECIST 1.1 in this context does not allow a statement to be made about tumor viability. For example, after microwave ablation, the ablation zone may be even larger than the original target lesion due to the safety margin maintained and the technical properties of the applicator, without, of course, indicating any progression.

### mRECIST

The modified RECIST criteria were developed specifically for HCC and only take into account the viable parts of the tumor, which are defined as regional contrast uptake in the arterial phase. Only the largest dimension of the contrast-enhancing component is measured. CR is present when there is a complete absence of arterial hyper-enhancement; PR when there is at least a 30% reduction in arterial hyper-enhancement; PD when there is at least a 20% increase in arterial hyper-enhancement or the appearance of new lesions, and SD is present when no significant change is measurable 5 26 .

The greatest advantage of using the classifications based on mRECIST criteria is that they capture and evaluate the morphological image correlate of the devascularizing therapies, namely the decline in the arterialized components.One limitation of this method is its lack of applicability in poorly vascularized tumors with ambiguous arterial hyper-enhancement, for example, under anti-angiogenic systemic treatment. In addition, inflammatory changes or bleeding can affect the contrast behavior and thus complicate the assessment of viability.

### EASL criteria

The European Association for the Study of the Liver’s criteria expand the mRECIST system by a two-dimensional measurement instead of the one-dimensional measurement of the contrast-enhancing tumor component in the axial plane and calculation of an area product. CR is present when contrast uptake is no longer detectable; PR occurs when there is at least a 50% reduction in the area of the contrast-enhancing portion; PD occurs when there is at least a 25% increase or the appearance of a new lesion, and SD is present in the absence of significant change 5 24 27 .

This classification based on EASL criteria enables a more differentiated assessment of irregularly-shaped lesions. However, it is methodologically more complex, potentially less reproducible, and difficult to standardize. For this reason, its application is limited largely to the context of studies.

### LI-RADS treatment response

The Liver Imaging Reporting and Data System Treatment Response Module (LI-RADS-TR), developed by the American College of Radiology, was widely updated in the 2024 version 21 22 23 . For the first time, a differentiation is made between radiation-associated (Radiation-TRA) and non-radiation-associated (Non-radiation-TRA) therapy-induced changes. In addition to the established categories “non-viable,” “viable,” and “non-evaluable,” the new category “non-progressing” was introduced in the Radiation-TRA, which describes persistent but not increasing contrast uptake after SBRT or SIRT. The definition of tumor viability has been simplified. The only decisive factor now is the presence of a “mass-like enhancement,” regardless of contrast agent phase. In addition, ancillary features (i.e. diffusion restriction and mild to moderate T2 hyperintensity) were integrated, which allow for optional upgrading of lesions. The current version therefore takes into account both the specific characteristics of radiation-induced changes and the requirements for standardized and reproducible reporting.

One key advantage of this classification system is that a standardized categorization is provided for each individual lesion. LI-RADS-TR, however, does not allow for quantification in percentages over time. This makes it less suitable in many cases for use in studies. It should also be noted that the LIRADS classification was explicitly developed only for high-risk patients with pre-existing liver cirrhosis or other risk factors for HCC. It is not intended for use in patients who do not have chronic liver disease.

**Fig. 1:**
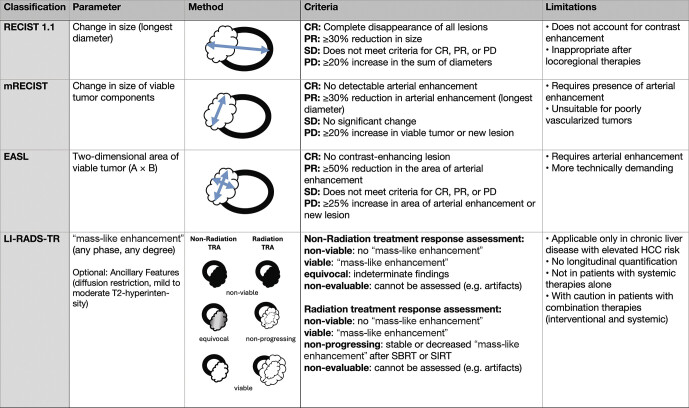
Overview of classification systems. CR: complete remission; PR: partial remission; SD: stable disease; PD: progressive disease; TRA: treatment response assessment; SIRT: selective internal radiation therapy; SBRT: stereotactic body radiotherapy.

A comparison summarizing the rating systems is shown in Fig. 1 .

## Image morphology according to therapy type

The interpretation of post-interventional imaging requires detailed understanding of treatment-specific morphology in order to differentiate between treatment effect, residual tumor viability, or recurrence and complications. The following section presents the methods already discussed with regard to their morphological image characteristics over time.

### Transarterial chemoembolization (TACE, DEB-TACE)

Post-therapeutic changes after TACE are generally caused by two factors: the ischemic effect of the embolic agent and the cytotoxic effect of the chemotherapeutic agent 28 . Differences, nevertheless, arise with regard to visualization of the TACE area in follow-up imaging due to the choice of therapeutic agent, as the Lipiodol used in conventional TACE remains in the liver tissue for a longer period of time and leads to corresponding changes that cannot be distinguished in DEB-TACE using loaded particles. MRI is superior to CT, particularly with regard to post-interventional imaging after conventional TACE 29 .

**Follow-up after TACE**

The ischemic and cytotoxic effect of TACE causes cell necrosis, which does not, however, lead directly to a reduction in the size of the target tumor. As a result, no reduction in size can be expected in the initial imaging after TACE, which is usually performed one to three months after therapy. In individual cases, however, a slight increase in size may occur due to the development of edema and bleeding 8 . Nevertheless, a decrease in the size of the treated tumor will be observed over time and leads to a decrease in diameter in the long term.

**Fig. 2:**
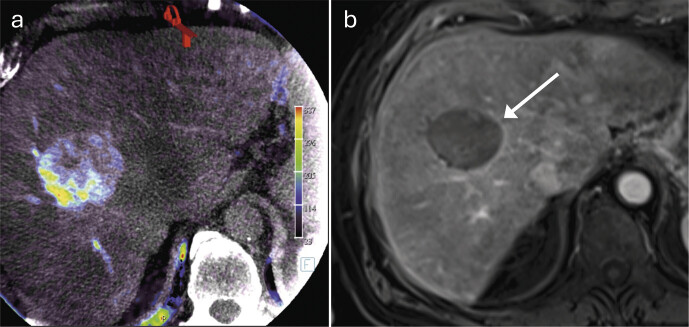
HCC in segment VIII in a 78-year-old patient. **a** Planning imaging using cone-beam CT prior to TACE. **b** The first follow-up MRI, performed Ten weeks after TACE, shows a thin peripheral enhancement (arrow) of the HCC in the late arterial phase of the fat-saturated T1-VIBE sequence, which is considered therapy-associated.

In contrast to the slow shrinkage of the target tumor, devascularization of a previously hypervascularized target tumor is observed immediately after TACE and should continue during further aftercare imaging 30 . While focal, intratumoral contrast enhancement detects residual tumor or local recurrence, a thin, hyperarterialized rim around the treated tumor is considered physiological ( Fig. 2 ) 31 . The underlying cause is inflammation, which occurs immediately after therapy and can persist for over a year. Peripherally located, wedge-shaped areas near the treated tumor are caused by the embolizing effect of TACE and correspond to the treated liver volume. These changes are particularly evident in the arterial phase as reduced contrast, while in later phases the contrast adjusts to that of the surrounding tissue. These described changes in perfusion resolve in the longer term after TACE 8 .

**Fig. 3:**
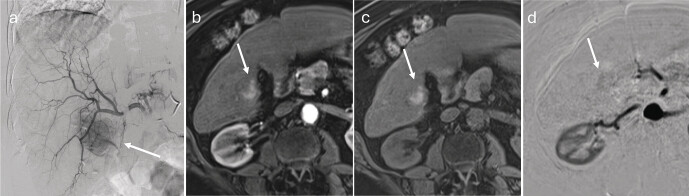
HCC in segment V in a 69-year-old female patient. **a** Pretherapeutic angiography prior to TACE of an HCC in segment V (arrow). **b** The first follow-up MRI, performed four weeks after therapy, shows the treated HCC as hyperintense (arrow) in the arterial phase of the fat-saturated T1-VIBE sequence. **c** However, this hyperintensity is already discernible on unenhanced images. **d** Subtraction images show no contrast enhancement. The hyperintensity is due to intratumoral hemorrhage or proteinaceous deposits within the tumor remnant and must not be misinterpreted as viable residual tumor.

The internal signal of a liver tumor treated with TACE appears heterogeneous in T1-weighted images and predominantly hypointense in T2-weighted images. However, T2-hyperintense areas can be caused by hemorrhages, liquid necrotic changes, or reactive edema, making it difficult to assess the treatment outcome based on signal behavior 32 . T1-hyperintense portions may occur as a result of hemorrhages or protein-rich deposits in the treated tumor. To distinguish these from residual tumor or recurrence in the arterial phase, subtraction images can be used ( Fig. 3 ).

As mentioned above, MRI is superior to CT in assessing the therapeutic effect after TACE, as it is not influenced by hardening artifacts caused by deposited Lipiodol. In addition, the opposed-phase T1 sequence can be used to assess the distribution of Lipiodol within the tumor 8 .

### Thermal ablation (RFA, MWA)

**Fig. 4:**
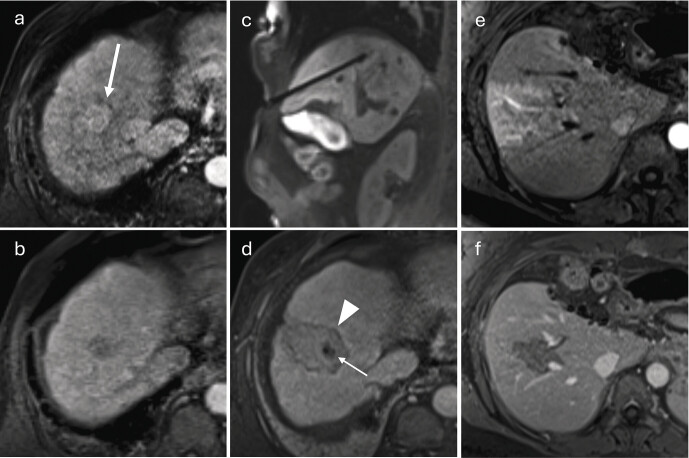
MRI-guided microwave ablation. **a** Planning imaging using fat-saturated T1-VIBE illustrates the HCC to be treated in segment VIII of a 72-year-old male patient, showing arterial hyper-enhancement (arrow). **b** The lesion exhibits washout in the late phase. **c** During the intervention, the applicator is advanced percutaneously under image guidance to the lesion (sagittal plane). **d** The final contrast-enhanced control imaging shows an ovoid ablation zone, hypo-enhancing relative to the untreated liver parenchyma (arrowhead). The non-viable lesion itself remains centrally visible within the ablation zone (arrow). **e** Due to the puncture, arteriovenous and arterioportal shunts may occur (different patient), which appear in the arterial phase as wedge-shaped peripheral hyper-enhancement adjacent to the ablation zone. **f** Shunts do not exhibit washout in the late phase.

The ablation area can be delineated in the MRI scan immediately after therapy. It is usually larger than the treated tumor, due to the safety margin of 5–10 mm and the minimum size of the therapy zone determined by the electrode or antenna 33 . In some cases, the previously treated lesion can still be delineated within the ablation zone for some time immediately after ablation ( Fig. 4 ). The internal signal for the ablation zone may be heterogeneous. T1-hyperintense areas often result from bleeding or protein-rich necrosis, whereas T2 hyperintensity results from edema, liquefaction, or tissue remodeling 34 .

**Follow-up after MWA/RFA**

The ablation zone typically has a narrow hyperperfused rim, which is due to post-interventional hyperemia and reactive inflammation, and it exhibits contrast enhancement without washout. In diffusion-weighted sequences, this shows reduced ADC values 35 . This pattern is considered physiological and persists in individual cases for several months 4 8 . In contrast, the occurrence of nodular, irregular contrast enhancement within or at the edge of the ablation area can be considered suspicious for residual tumor. Subtraction images from the arterial and native phase can also help to differentiate true arterial enhancement from hemorrhages in cases of ambiguous hyperintensity 32 . In the authors’ experience, DWI is only partially suitable for assessing the central ablation zone. Although recurrences with reduced ADC values and necrosis with increased ADC values have been described 36 , the signal can vary greatly.

**Fig. 5:**
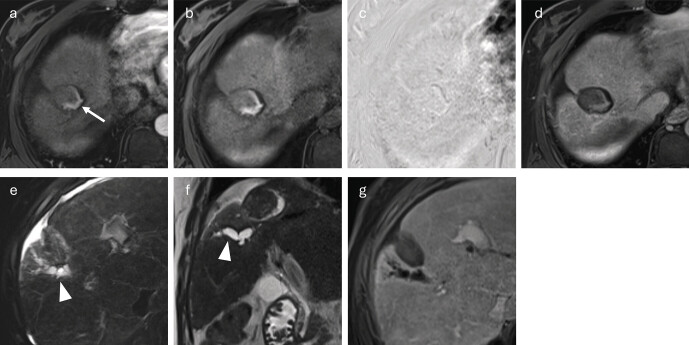
Follow-up imaging after microwave ablation. **a** At 6-week follow-up after microwave ablation, the ablation zone already shows a reduction in size. In the contrast-enhanced T1-VIBE arterial phase, hyperintense structures are visible within the ablation zone (arrow). **b** The hyperintensity is also discernible in the unenhanced phase and can be attributed to hemorrhage and proteinaceous deposits. **c** Subtraction imaging confirms that these are not vascularized components. **d** The ablation area remains hypointense compared to the untreated liver parenchyma in the portal venous phase. **e** At a later follow-up twelve weeks after therapy, dilated bile ducts (arrowhead) can be delineated peripheral to the ablation zone (T2-weighted sequence). These are due to thermal injury to the bile ducts and are not indicative of cholestasis caused by tumor recurrence. Such damage is irreversible. **f** Coronal T2-weighted sequence. The dilated bile ducts appear hyperintense (arrowhead). **g** In the contrast-enhanced T1-weighted sequence, the bile ducts appear hypointense.

**Fig. 6:**
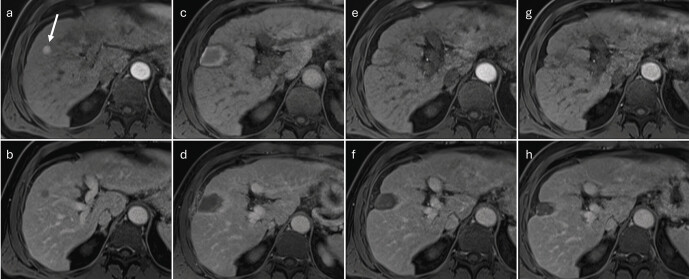
Long-term course after percutaneous microwave ablation of an HCC in segment V. **a** Fat-saturated T1-VIBE, arterial phase: focal HCC with arterial phase hyper-enhancement (arrow). **b** Delayed phase: washout of the lesion. **c** First follow-up at four weeks, arterial phase: ablation zone extending to the liver capsule; central T1 hyperintensities (hemorrhage/protein-rich necrosis); no residual arterial phase hyper-enhancement. **d** First follow-up at four weeks, delayed phase: hypocontrasted ablation zone with a thin, reactive hyperemic rim. **e** Ten months after ablation, arterial phase: marked volume reduction of the ablation zone; central hyperintensities regressing. **f** Ten months, delayed phase: clear delineation of the hypocontrasted area; early capsular retraction. **g** Twenty-eight months, arterial phase: no evidence of viable tumor. **h** Twenty-eight months, delayed phase: further shrinkage of the ablation scar and progressive capsular retraction.

Immediate post-interventional, perihepatic fluid collections can occur, particularly in quite peripheral lesions as a reaction of the liver capsule to thermal injury, and they are spontaneously regressive. However, it is important to differentiate this fluid buildup from relevant bleeding after retracting the electrode or applicator. Individual air figures in the ablation zone are typically visible immediately after the intervention. These can persist for up to two weeks after therapy, are considered physiological, and should not be interpreted as an infection. Wedge-shaped, hypervascularized areas visible immediately after ablation correspond to arteriovenous or arterioportal shunts resulting from the puncture ( Fig. 4 **e** , **f** ). These perfusion changes can persist for weeks to months. In contrast to these vascular changes, subsegmental bile duct dilations may occur peripherally to the ablation zone within a few weeks after ablation. These bile duct changes are irreversible, but they are not a sign of recurrent cholestasis. Rather they are due to thermal damage to the bile ducts ( Fig. 5 ). Over months and years, the ablation zone shrinks and eventually persists as a scarred, low-signal residue ( Fig. 6 ).

### SIRT (selective internal radiotherapy)

**Fig. 7:**
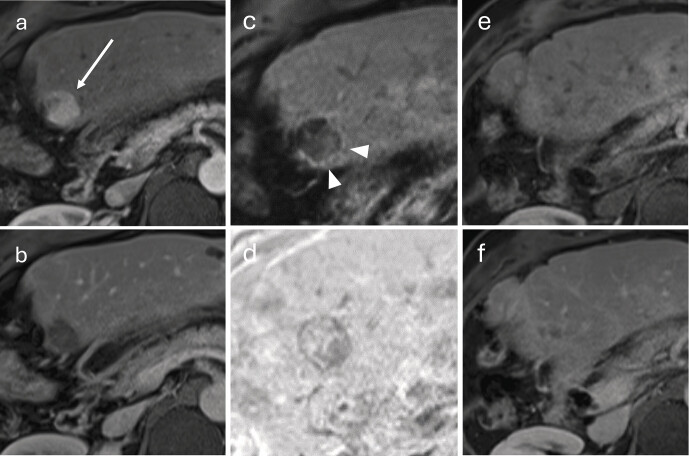
Follow-up after SIRT of an HCC in liver segment III in a 53-year-old patient (status post right hemihepatectomy). **a** Pre-treatment, fat-saturated T1-VIBE, arterial phase: hypervascular HCC adjacent to the resection margin (arrow). **b** Pre-treatment, delayed phase: washout of the lesion. **c** Twelve weeks after SIRT, arterial phase: thin peripheral rim enhancement around the treated lesion (arrowheads), consistent with post-therapeutic hyperemia. **d** Twelve weeks, subtraction: persistent central enhancement; after SIRT, central arterial hyper-enhancement may persist for weeks to months and should not be misinterpreted as early treatment failure. **e** Nine months after SIRT, arterial phase: no arterial hyper-enhancement detectable. **f** Nine months, delayed phase: subtle residual scar.

After SIRT, the morphological response to treatment sets in with a delay after three to six months 14 37 . Arterial hyper-enhancement with or without tumor washout can therefore persist without indicating treatment failure ( Fig. 7 ) 38 . In the early weeks after therapy, inhomogeneous signal changes are often seen in MRI in T1- and T2-weighted sequences. T1 hyperintensities occur due to blood degradation products or protein precipitates; T2 hyperintensities occur due to necrosis or edema. Initially, the tumor volume often remains stable or even increases slightly over the first four months due to inflammatory reactions or intratumoral bleeding (pseudoprogression) 8 . A narrow enhancement rim (typically < 5 mm) at the edge of the area occurs in up to 50% of cases within half a year and should not be misinterpreted as tumor recurrence due to its circular configuration 32 39 . Nodular arterial contrast enhancement may persist even after three months in successfully treated tumors, but may regress over time 8 . Granulation tissue can also potentially appear in this form and can often only be differentiated through further follow-up. Occasionally, radiation-induced changes occur in the surrounding parenchyma, recognizable as geographic wedge-shaped areas with transient enhancement changes that may persist for up to six months or longer and do not represent an infiltrative tumor. Fibrosis, with possible capsular retraction and volume reduction, appears after months. This effect is particularly pronounced in radiation lobectomy, which, in addition to atrophy of the treated liver lobe, leads to a compensatory hypertrophy of the untreated liver 40 .

### Stereotactic radiotherapy (SBRT)

**Fig. 8:**
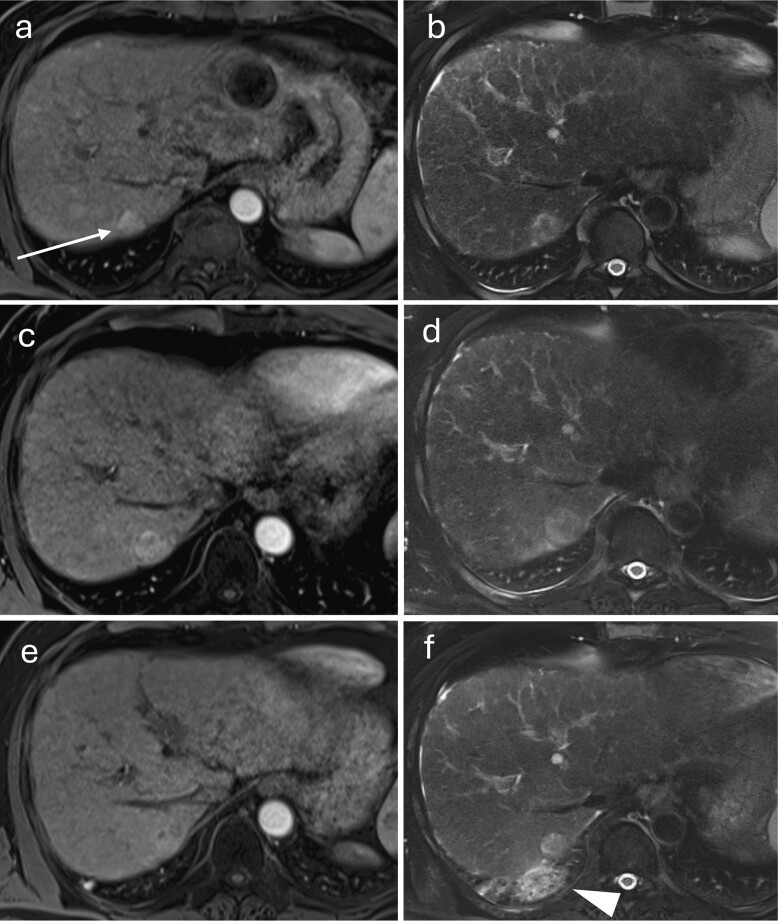
Follow-up after SBRT of a subcapsular HCC in liver segment VII. **a** A subcapsular HCC in liver segment VII (arrow) appears hyperarterialized on the fat-saturated T1-VIBE sequence prior to therapy. **b** The lesion shows mild hyperintensity on the fat-saturated T2-weighted sequence. **c** At 6-week follow-up after SBRT, the lesion shows slight progression in size. **d** Peritumoral edematous changes are visible within the previous radiation field (“focal liver reaction”). **e** At further follow-up performed twelve weeks after SBRT, the arterial enhancement of the lesion has markedly regressed. **f** Peritumoral signal alterations have also decreased. The T2-hyperintense signal changes in the right lower lung lobe are due to radiation pneumonitis (arrowhead).

**Fig. 9:**
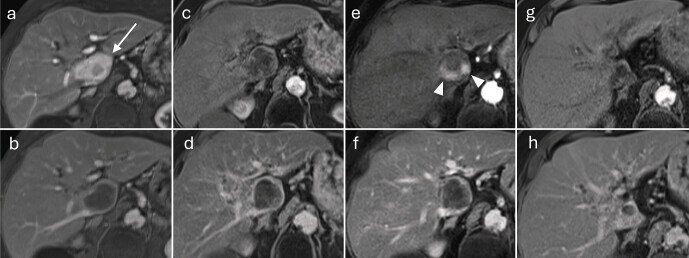
SBRT and TACE of an extensive HCC in segment I in a 54-year-old woman. **a** Pre-treatment, fat-suppressed T1-VIBE, arterial phase: extensive hypervascular, histologically confirmed HCC in segment I (arrow). **b** Pre-treatment, delayed phase: washout of the lesion. **c** Six months after SBRT, arterial phase: no arterial hyper-enhancement detectable; no relevant change in lesion size. **d** Six months after SBRT, delayed phase: tumor size remains unchanged. **e** Ten months after SBRT, arterial phase: new nodular arterialized foci along the posterior circumference of the lesion (arrowheads) indicating recurrence; TACE recommended by the multidisciplinary tumor board. **f** Ten months, delayed phase: still no relevant size change. **g** Seven months after successful TACE (17 months after SBRT overall), arterial phase: no arterially enhancing lesions suspicious for recurrence. **h** Seven months after successful TACE, delayed phase: marked reduction in lesion size.

The morphological changes after SBRT also occur with a delay ( Fig. 8 , Fig. 9 ). In imaging up to about three months after therapy, the tumor size usually remains unchanged or may increase slightly 38 . In up to 75% of cases, arterial hyper-enhancement persists for several months, even in cases of histologically confirmed necrosis, and only regresses significantly after about six to twelve months 20 . Occasionally, a thin marginal enhancement occurs, which is less frequent and less pronounced than in SIRT. This enhancement is also caused by inflammation and usually regresses within six months. In parallel, edematous changes develop in the peritumoral liver tissue that can persist for up to a year or even longer; these changes are often referred to as focal liver reaction (FLR), and they are detectable as a geographically configured T2-hyperintense area with arterial hyper-enhancement and possible hypo-enhancement in the portal venous phase 20 38 . Completely necrotic tumor parts show increased ADC values in diffusion-weighted sequences (Navin et al., 2022). After about six months, fibrotic changes increasingly develop, which persist in the long term and can be accompanied by significant capsular retraction.

**Fig. 10:**
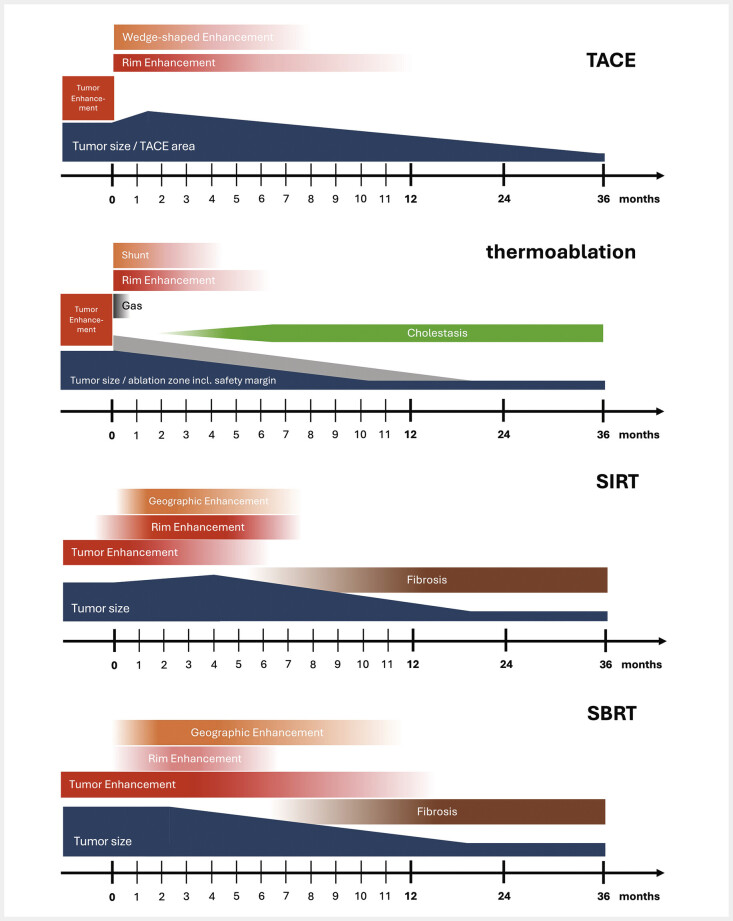
Sequence of therapy-associated changes over time after interventional treatment.

Fig. 10 illustrates the therapy-associated changes over time after interventional treatment.

## Aftercare recommendations and follow-up protocols

Recommendations for imaging follow-up after locoregional therapy of HCC are contained in both the German S3 guideline and the European EASL Clinical Practice Guideline. Both guidelines prefer the use of multiphasic dynamic MRI, with contrast-enhanced CT examinations as a valid alternative 3 41 .

According to the S3 guideline, the initial imaging follow-up after thermoablation, SIRT, and TACE should take place between four and twelve weeks; for SBRT, the first follow-up is recommended after twelve weeks, at the earliest. The aim of these initial checks is to assess the technical success and to detect residual tumor tissue.

In the continued course of the first year, the S3 guideline recommends regular imaging checks at intervals of approximately three months. This close-meshed approach is justified by the significantly increased risk of recurrence within the first year, which is about 6.5 times higher than in later periods 42 .

In the second year, the follow-up interval can be extended to three to six months. The S3 guideline recommends an aftercare period of at least two years, although the continuation may be longer depending on the individual risk of recurrence and patient profile. At the authors’ institution, it is common to extend aftercare to five years.

After completion of the aftercare phase, patients should be transferred to regular ultrasound screening with semi-annual check-ups according to both guidelines. This recommendation applies particularly to patients with existing liver cirrhosis or an increased risk of recurrence due to chronic liver disease.

## Summary

Imaging plays a central role in the post-interventional assessment of treatment response in hepatocellular carcinoma. The type and temporal dynamics of the image morphological changes vary depending on the type of therapy used. After thermoablation, an ablation zone immediately appears, which shrinks over time. After successful TACE, there is an immediate reduction in arterial hyper-enhancement, but the impacts of cytotoxic effects only become apparent after weeks. However, after SIRT and SBRT, morphological changes occur with a delay. Initial size stability should not be confused with a lack of response to treatment. Key elements of the radiology assessment are arterial contrast enhancement and washout. It can also be helpful to use subtraction images. Understanding typical pitfalls, such as post-interventional inflammatory hyperemia, is critical and can help to avoid misinterpretations.

Imaging should be based on standardized aftercare plans such as mRECIST or LI-RADS-TR, which interpret the viable parts of the tumor. Looking strictly at morphological size changes, such as in the context of RECIST 1.1, does not adequately reflect treatment success. The version of LI-RADS-TR updated in 2024 includes the category “non-progressing” and for the first time takes radiation-associated changes into account; it also simplifies the definition of tumor viability. In addition, ancillary features such as diffusion restriction or T2 hyperintensity enable more differentiated classification of ambiguous findings and help to provide a standardized assessment.

The limitations of this review result from the heterogeneity of the post-interventional findings, as not all of the changes described occur in every case, their development over time can vary greatly, and combination therapies can also have an effect. The impact of systemic treatments was not considered in this study, which limits its transferability to multimodal therapy concepts.
