# Supplementary material for: MRI after Interventional Therapy of Hepatocellular Carcinoma: Typical Changes over Time
Source: Rofo. 2025 Nov 21;198(5):610–23. doi: 10.1055/a-2724-6488 (PMC13120846; doi:10.1055/a-2724-6488)
Supplement: Related texts

# Review article: Bildgebung nach interventioneller Therapie des hepatozellulären Karzinoms: typische MRT-Veränderungen im zeitlichen Verlauf

## Abstract

**Hintergrund:**

Die Magnetresonanztomografie (MRT) stellt das bevorzugte Verfahren zur bildgebenden Verlaufskontrolle nach interventioneller Therapie des hepatozellulären Karzinoms (HCC) dar. Ziel dieser Übersichtsarbeit ist es, die charakteristischen zeitabhängigen MRT-Veränderungen nach transarterieller Chemoembolisation (TACE), Radiofrequenz- und Mikrowellenablation (RFA, MWA), selektiver interner Radiotherapie (SIRT) und stereotaktischer Strahlentherapie (SBRT) darzustellen sowie die Bedeutung standardisierter Klassifikationssysteme (mRECIST, EASL, LI-RADS-TR) für eine strukturierte Befundung zu erläutern.

**Methode:**

Für diesen Artikel wurde eine strukturierte Literaturrecherche in PubMed für den Zeitraum 2000 bis 2025 durchgeführt. Als Suchbegriffe wurden „hepatocellular carcinoma“, „magnetic resonance imaging“, „thermal ablation“, „transarterial chemoembolization“, „transarterial radioembolization“, „stereotactic body radiotherapy“ und „treatment response“ verwendet. Ergänzend wurden aktuelle nationale und internationale Leitlinien berücksichtigt sowie hausinterne Erfahrungswerte in die Darstellung einbezogen.

**Ergebnisse:**

Postinterventionelle Veränderungen in der MRT variieren je nach eingesetztem Therapieverfahren. Nach Thermoablation und TACE treten typische morphologische Veränderungen unmittelbar auf, wohingegen sich Therapieeffekte der SIRT und SBRT erst nach Wochen bis Monaten eindeutig manifestieren. Eine fundierte Kenntnis der standardisierten Bewertungssysteme mRECIST, EASL und LI-RADS-TR ist essenziell, um eine präzise und strukturierte Bewertung des Therapieansprechens sicherzustellen.

**Schlussfolgerung:**

Die Kenntnis spezifischer MRT-Befunde und standardisierter Bewertungssysteme ist entscheidend für die strukturierte Nachsorge des hepatozellulären Karzinoms nach interventioneller Therapie. Limitationen ergeben sich aus der Heterogenität der Befunde, variablen zeitlichen Verläufen sowie möglichen Einflüssen von Kombinationstherapien. Zudem wurden Systemtherapien nicht berücksichtigt, wodurch die Übertragbarkeit eingeschränkt ist.

**Kernaussagen:**

- Die Magnetresonanztomografie ist für die Beurteilung des Therapieansprechens und zur Früherkennung von Rezidiven nach interventioneller Therapie des hepatozellulären Karzinoms unerlässlich.
- Morphologische Veränderungen nach Therapie sind verfahrensabhängig und zeigen deutliche Unterschiede im zeitlichen Verlauf. SIRT und SBRT zeigen verzögerte Veränderungen, während TACE und Thermoablation unmittelbare Effekte aufweisen.
- Standardisierte Klassifikationssysteme (mRECIST, EASL, LI-RADS-TR) ermöglichen eine strukturierte und reproduzierbare Bewertung des Therapieansprechens.
- Die Kenntnis charakteristischer Befunde und potenzieller Fallstricke ist entscheidend, um Residualtumor, Rezidiv und therapiebedingte Veränderungen zuverlässig zu unterscheiden.
- Die Arbeit leistet einen Beitrag zum aktuellen Wissensstand, indem sie die vorhandene Evidenz systematisch zusammenführt und praxisorientiert im Kontext der MRT-Bildgebung nach interventioneller Therapie darstellt.

**Zitierweise:**

- Kübler J, Ashkar A, Winkelmann MT et al. MRI after Interventional Therapy of Hepatocellular Carcinoma: Typical Changes over Time. Rofo 2025; DOI 10.1055/a-2724-6488

## Abkürzungen

ADC: Apparent Diffusion Coefficient
AFP: Alpha-Fetoprotein
BCLC: Barcelona Clinic Liver Cancer
CR: Complete Response
CT: Computertomografie
cTACE: Conventional Transarterial Chemoembolization
DEB-TACE: Drug-Eluting Bead Transarterial Chemoembolization
DWI: Diffusion-Weighted Imaging
EASL: European Association for the Study of the Liver
FLR: Focal Liver Reaction
HCC: Hepatozelluläres Karzinom
INR: International Normalized Ratio
LI-RADS-TR: Liver Imaging Reporting and Data System – Treatment Response
mRECIST: Modified Response Evaluation Criteria In Solid Tumors
MRT: Magnetresonanztomografie
MWA: Mikrowellenablation
PD: Progressive Disease
PR: Partial Response
RFA: Radiofrequenzablation
RS: Radiation Segmentectomy
RL: Radiation Lobectomy
SBRT: Stereotactic Body Radiotherapy
SD: Stable Disease
SIRT: Selektive interne Radiotherapie
TACE: Transarterial Chemoembolization
TRA: Treatment Response Assessment
US: Ultraschall
VIBE: Volumetric Interpolated Breath-hold Examination

## Einleitung

Die interventionelle Radiologie hat sich in den letzten zwei Jahrzehnten zu einem integralen Bestandteil der multimodalen Therapie des hepatozellulären Karzinoms (HCC) entwickelt. Insbesondere bei nicht resektablen Tumoren stellen lokale Therapieverfahren wie die thermische Ablation mittels Radiofrequenzablation (RFA) oder Mikrowellenablation (MWA), die transarterielle Chemoembolisation (transarterial chemoembolization, TACE) sowie die selektive interne Radiotherapie (SIRT) wesentliche Behandlungsoptionen dar 1 . Ergänzt wird dieses Spektrum zunehmend durch hochpräzise perkutane Strahlentherapie (stereotactic body radiotherapy, SBRT) 2 .

Die Bildgebung spielt in allen Phasen der interventionellen Therapie eine zentrale Rolle: in der initialen Therapieplanung, zur postprozeduralen Erfolgskontrolle, zum Ausschluss von Residualtumor sowie in der strukturierten Nachsorge zur frühen Detektion eines Progresses. Unter den bildgebenden Verfahren gilt die Magnetresonanztomografie (MRT) als Methode der Wahl, da sie im Vergleich zur Computertomografie eine höhere Sensitivität bei gleichwertiger Spezifität, insbesondere für Läsionen unter 2 cm, bietet. Durch ihren überlegenen Weichteilkontrast ermöglicht sie eine präzisere Läsionscharakterisierung und eignet sich somit besonders für die postinterventionelle Verlaufskontrolle 3 . Dabei erfordern sowohl die Beurteilung des morphologischen Ansprechens als auch das Erkennen therapiebedingter Veränderungen detaillierte Kenntnisse der behandlungsspezifischen Bildmuster 4 5 6 7 .

Standardisierte Klassifikationssysteme wie mRECIST, EASL und das Treatment Response-Modul der LI-RADS-Klassifikation bieten strukturierte Kriterien zur Beurteilung des radiologischen Therapieansprechens. Jedes der Klassifikationssysteme hebt sich durch unterschiedliche Stärken hervor, jedoch sollten auch die jeweiligen Limitationen der Aussagekraft bekannt sein 5 8 9 .

Ziel dieses Übersichtsartikels ist es, die wesentlichen Aspekte der postinterventionellen Magnetresonanztomografie nach lokalen Therapieverfahren beim hepatozellulären Karzinom darzustellen.

## Methoden

Für diese Übersichtsarbeit wurde eine strukturierte Literaturrecherche in PubMed durchgeführt, die den Zeitraum von Januar 2000 bis März 2025 abdeckte. Eingesetzt wurden die Suchbegriffe „hepatocellular carcinoma“, „magnetic resonance imaging“, „thermal ablation“, „transarterial chemoembolization“, „transarterial radioembolization“, „stereotactic body radiotherapy“ und „treatment response“. Eingeschlossen wurden englisch- und deutschsprachige Originalarbeiten und Übersichtsarbeiten zur postinterventionellen Bildgebung beim HCC. Fallberichte wurden ausgeschlossen. Insgesamt wurden 1689 Publikationen identifiziert. Nach Entfernung von Duplikaten und dem Ausschluss thematisch nicht passender Arbeiten verblieben 281 Artikel. Nach Screening der Abstracts wurden 193 Arbeiten in die engere Auswahl genommen. Davon wurden 98 im Volltext geprüft. Zusätzlich wurden aktuelle nationale und internationale Leitlinien berücksichtigt sowie hausinterne klinische Erfahrungswerte eingebracht.

## Interventionelle Therapieverfahren

## Transarterial Chemoembolization (TACE, DEB-TACE)

Die TACE stellt die empfohlene Standardtherapie für Patientinnen und Patienten mit HCC im intermediären Stadium (BCLC B) dar, sofern kurative Optionen wie Resektion, Transplantation oder Ablation nicht möglich sind. Voraussetzung ist eine erhaltene Leberfunktion (Child-Pugh A bis B7) und das Fehlen extrahepatischer Metastasen oder makroskopischer Gefäßinvasion 3 . Die Grundlage des Verfahrens ist die vorwiegend arterielle Versorgung des HCC, während das angrenzende Lebergewebe portalvenös versorgt wird. Die TACE wird bevorzugt selektiv durchgeführt und kann als conventional TACE (cTACE) angewendet werden, bei der ein Chemotherapeutikum mit Lipiodol als Trägermedium intraarteriell appliziert wird. Alternativ werden bei der DEB-TACE (drug-eluting bead Transarterial Chemoembolization) mit Medikamenten beladene Embolisationspartikel appliziert. Ziel ist neben einer Ischämie-induzierten Tumornekrose ein lokaler zytostatischer Effekt bei geringer systemischer Toxizität. Der embolische Effekt ist unmittelbar nach Therapie vorhanden und als fehlendes arterielles Hyperenhancement in der Bildgebung abgrenzbar.

## Thermische Ablationen (RFA, MWA)

Die thermische Ablation mittels Radiofrequenzablation (RFA) oder Mikrowellenablation (MWA) stellt eine kurative Therapieoption für Patientinnen und Patienten mit frühem hepatozellulärem Karzinom (BCLC 0 und A) dar, insbesondere bei Tumoren mit einem Durchmesser bis zu drei Zentimetern und bei limitierter Anzahl von Läsionen. Voraussetzung ist eine erhaltene Leberfunktion und das Fehlen makroskopischer Gefäßinvasion oder extrahepatischer Metastasierung 3 . Die Radiofrequenzablation (RFA) und Mikrowellenablation (MWA) unterscheiden sich in ihrem technischen Ansatz, verfolgen jedoch beide das Ziel der irreversiblen Koagulationsnekrose des Tumors durch lokal induzierte Hyperthermie. Während die RFA auf einem Wechselstrom im Bereich von 400–500 kHz basiert, werden bei der MWA elektromagnetische Wellen mit Frequenzen im Bereich von 915 MHz oder 2,45 GHz verwendet. Im Vergleich zur RFA bietet die MWA eine höhere Energiepenetration, kürzere Applikationszeiten und eine geringere Anfälligkeit gegenüber dem sogenannten „heat sink effect“, welcher den ungewollten Wärmeabtransport durch große Gefäße in der Nähe der Zielläsion bezeichnet 10 11 . Die thermisch induzierte Nekrosezone ist in der MRT-Bildgebung unmittelbar nach Therapie abgrenzbar und weist im längerfristigen Verlauf eine Größenregredienz auf.

## Selektive Interne Radiotherapie (SIRT)

Die selektive interne Radiotherapie (SIRT), auch transarterielle Radioembolisation (TARE) genannt, wird bei Patientinnen und Patienten mit HCC in fortgeschrittenem oder intermediärem Stadium eingesetzt, insbesondere wenn andere lokoregionäre Verfahren nicht geeignet oder erschöpft sind 3 12 . β-Strahler-emittierende Mikrosphären, meist Yttrium-90, werden selektiv über die arterielle Tumorversorgung appliziert. Aufgrund der geringen Penetrationstiefe der Strahlung von im Mittel 2,4 mm kann selektiv behandelt und umliegendes Gewebe geschont werden 13 . Prätherapeutisch erfolgt eine angiografische Simulation mit Technetium-99m-MAA zum Ausschluss von pulmonalen oder gastrointestinalen Shunts, sodass unter Therapie keine ungewollte extrahepatische Anreicherung erfolgt. Indikationen bestehen bei portalvenöser Tumorinvasion ohne dekompensierte Leberzirrhose sowie bei nicht resektablen Tumoren mit erhaltener Leberfunktion (in der Regel Child-Pugh A bis B7). Ursprünglich als palliative Maßnahme bei fortgeschrittener Erkrankung eingesetzt, wird die TARE heute zunehmend zur Bridging-Therapie vor Lebertransplantation, zur Downstaging-Therapie, als kurative Therapie im Rahmen der Radiation Segmentectomy (RS) und zur Radiation Lobectomy (RL) zur Vorbereitung auf eine Resektion angewandt 14 . Der embolische Effekt steht gegenüber der Strahlenschädigung im Hintergrund, sodass sich posttherapeutische Veränderungen in der Bildgebung erst im längerfristigen Verlauf zeigen 15 .

## Stereotaktische Strahlentherapie (SBRT)

Die stereotaktische Strahlentherapie (SBRT) ist nach aktueller S3-Leitlinie eine nicht invasive Therapieoption für Patientinnen und Patienten, wenn thermische Ablation oder Resektion technisch nicht durchführbar oder kontraindiziert sind oder im Falle eines Rezidivs nach thermischer Ablation 3 . Sie kann in verschiedenen Stadien des HCC zum Einsatz kommen 3 16 17 . Darüber hinaus wird die SBRT ebenfalls zunehmend als Bridging- oder Downstaging-Strategie vor Lebertransplantation genutzt. SBRT ermöglicht durch präzise Dosisapplikation eine gezielte Tumornekrose bei bestmöglicher Schonung des umliegenden Parenchyms 18 . Die pathophysiologischen Effekte von SBRT beruhen sowohl auf direkten DNA-Doppelstrangbrüchen in Tumorzellen als auch auf sekundären biologischen Prozessen wie endothelialer Schädigung und einer Modulation des tumorassoziierten Mikromilieus, die immunvermittelte antitumorale Effekte fördern kann 19 . Da diese Prozesse verzögert ablaufen, kommt es erst im Verlauf zu bildmorphologisch fassbaren Veränderungen 20 .

## Klassifikationssysteme zur Therapieevaluation

Die Bewertung des Therapieansprechens nach lokoregionären Verfahren beim hepatozellulären Karzinom erfordert spezifische Klassifikationssysteme, da eine klassische Größenvermessung nach Response Evaluation Criteria in Solid Tumors (RECIST) einen Therapieerfolg oder ein Therapieversagen nur unzureichend abbildet. Entscheidend für die Beurteilung der Tumorvitalität ist die Evaluation des Kontrastmittelverhaltens, insbesondere der arteriellen Hypervaskularisation und des Washout-Phänomens. Zur standardisierten Befundung wurden verschiedene Systeme wie die modifizierten RECIST (mRECIST), die European Association for the Study of the Liver (EASL)-Kriterien und der Liver Imaging Reporting and Data System Treatment Response (LI-RADS-TR)-Algorithmus entwickelt, die jeweils unterschiedliche Schwerpunkte und Limitationen aufweisen. Mit der Version 2024 wurde das LI-RADS-Treatment-Response-Assessment (TRA) grundlegend überarbeitet. Erstmals wird zwischen einem Non-Radiation-TRA-Core für Ablations- und Embolisationstherapien sowie einem Radiation-TRA-Core für strahlenbasierte Verfahren (SIRT, SBRT) unterschieden 21 22 23 . Eine strukturierte Anwendung dieser Systeme ist entscheidend für die valide Therapiekontrolle und die weitere klinische Entscheidungsfindung 5 8 24 .

## RECIST 1.1

Die Response Evaluation Criteria in Solid Tumors in der Version 1.1 definieren Therapieansprechen ausschließlich über Größenveränderungen messbarer Läsionen (größter Längsdurchmesser). Ein vollständiges Ansprechen (Complete Response, CR) entspricht dem Verschwinden aller Läsionen, eine partielle Remission (PR) erfordert eine mindestens 30%ige Durchmesserreduktion, während ein Progress (PD) als Zunahme ≥20% oder Auftreten neuer Läsionen interpretiert wird. Ein stabiler Befund liegt vor, wenn die Größenänderung keiner CR, PR oder PD entspricht 25 .

Nach lokoregionärer Therapie des HCC verbleiben jedoch häufig avaskuläre, aber morphologisch persistierende Läsionen, sodass RECIST 1.1 in diesem Kontext keine Aussage über Tumorvitalität erlaubt. Beispielsweise stellt sich nach Mikrowellenablation die Ablationszone durch Einhaltung eines Sicherheitssaums und technische Eigenschaften des Applikators sogar größer als die ursprüngliche Zielläsion dar, natürlich ohne dass dadurch ein Progress vorliegt.

## mRECIST

Die modifizierten RECIST wurden speziell für das HCC entwickelt und berücksichtigen ausschließlich den vitalen Tumoranteil, definiert als regionale Kontrastmittelaufnahme in der arteriellen Phase. Es wird nur die größte Dimension der KM-aufnehmenden Komponente gemessen. CR liegt vor bei einem vollständigen Fehlen von arteriellem Hyperenhancement. PR bei einer mindestens 30%igen Reduktion des arteriellen Hyperenhancements, PD bei zumindest 20%iger Zunahme des arteriellen Hyperenhancements oder Auftreten neuer Läsionen. SD liegt vor, wenn keine signifikante Änderung messbar ist 5 26 .

Der größte Vorteil der Klassifikation nach mRECIST besteht darin, dass das bildmorphologische Korrelat der devaskularisierenden Therapien, nämlich der Rückgang der arterialisierten Anteile, erfasst und bewertet wird. Eine Limitation der Methode ist jedoch die fehlende Anwendbarkeit bei schlecht vaskularisierten Tumoren mit uneindeutigem arteriellem Hyperenhancement, etwa unter antiangiogener Systemtherapie. Zudem können inflammatorische Veränderungen oder Einblutungen das Kontrastverhalten beeinflussen und so die Vitalitätsbeurteilung erschweren.

## EASL-Kriterien

Die European Association for the Study of the Liver-Kriterien erweitern die mRECIST-Systematik durch eine zweidimensionale Messung anstelle der eindimensionalen Messung der kontrastmittelaufnehmenden Tumorkomponente in der axialen Ebene und Errechnung eines Flächenproduktes. Eine CR liegt vor, wenn keine Kontrastaufnahme mehr nachweisbar ist. Eine PR liegt bei einer zumindest 50%igen Flächenreduktion des kontrastmittelaufnehmenden Anteils vor und eine PD bei einer zumindest 25%igen Zunahme oder Auftreten einer neuen Läsion. SD liegt bei fehlender signifikanter Änderung vor 5 24 27 .

Diese Klassifikation nach EASL erlaubt eine differenziertere Beurteilung bei irregulär geformten Läsionen. Sie ist jedoch methodisch aufwendiger, potenziell weniger reproduzierbar und schwer standardisierbar und beschränkt sich in ihrer Anwendung daher weitgehend auf Studienkontexte.

## LI-RADS Treatment Response

Das Liver Imaging Reporting and Data System Treatment Response-Modul (LI-RADS-TR), entwickelt vom American College of Radiology, wurde in der Version 2024 umfassend aktualisiert 21 22 23 . Erstmals erfolgt eine Differenzierung zwischen strahlenassoziierten (Radiation-TRA) und nicht-strahlenassoziierten (Non-radiation-TRA) therapiebedingten Veränderungen. Neben den etablierten Kategorien „non-viable“, „viable“ und „non-evaluable“ wurde im Radiation-TRA die neue Kategorie „non-progressing“ eingeführt, welche persistierende, aber nicht zunehmende Kontrastmittelaufnahmen nach SBRT oder SIRT beschreibt. Die Definition der Tumorvitalität wurde vereinfacht. Maßgeblich ist nun allein das Vorliegen eines „masslike enhancement“ unabhängig von der Kontrastmittelphase. Ergänzend wurden sogenannte ancillary features (Diffusionsrestriktion und milde bis moderate T2-Hyperintensität) integriert, die eine optionale Aufstufung von Läsionen ermöglichen. Damit berücksichtigt die aktuelle Version sowohl die Besonderheiten strahleninduzierter Veränderungen als auch die Anforderungen an eine standardisierte und reproduzierbare Befundung. Ein entscheidender Vorteil dieses Klassifikationssystems ist, dass für jede einzelne Läsion eine standardisierte Kategorisierung vorgesehen ist. LI-RADS-TR erlaubt jedoch keine prozentuale Verlaufsquantifizierung. Damit ist sie für Studienzwecke in vielen Fällen weniger geeignet. Zu beachten ist außerdem, dass die LIRADS-Klassifikation explizit nur für Risikopatienten mit vorbestehender Leberzirrhose oder anderen Risikofaktoren für HCC entwickelt worden ist. Sie ist nicht vorgesehen für die Verwendung bei Patientinnen und Patienten ohne chronische Lebererkrankung.

Eine synoptische Gegenüberstellung der Bewertungssysteme findet sich in Abb. 1 .

**Abb. 1:** Übersicht über Klassifikationssysteme. CR: Komplettremission, PR: Partielle Remission, SD: Stabile Erkrankung, PD: Progrediente Erkrankung, TRA: Treatment Response Assessment, SIRT: Selektive interne Radiotherapie, SBRT: Stereotactic Body Radiotherapy.

## Bildmorphologie nach Therapieform

Die Interpretation der postinterventionellen Bildgebung erfordert ein profundes Verständnis der behandlungsspezifischen Morphologie, um zwischen Therapieeffekt, residueller Tumorvitalität bzw. Rezidiv und Komplikationen differenzieren zu können. Nachfolgend werden die bereits erläuterten Verfahren hinsichtlich ihrer bildmorphologischen Charakteristika im zeitlichen Verlauf dargestellt.

## Transarterielle Chemoembolisation (TACE, DEB-TACE)

Die posttherapeutischen Veränderungen nach TACE werden allgemein durch zwei Faktoren verursacht: den ischämischen Effekt durch das Embolisat sowie den zytotoxischen Effekt durch das Chemotherapeutikum 28 . Unterschiede in der Darstellung des TACE-Areals im bildgebenden Follow-up ergeben sich dennoch durch die Wahl des Therapeutikums, da das bei der konventionellen TACE applizierte Lipiodol über einen längeren Zeitraum im Lebergewebe verbleibt und zu entsprechenden Veränderungen führt, die bei der DEB-TACE mit beladenen Partikeln nicht abgrenzbar sind. Insbesondere hinsichtlich der postinterventionellen Bildgebung nach konventioneller TACE ist die MRT der CT überlegen 29 .

**Verlauf nach TACE**

Der ischämische und zytotoxische Effekt der TACE verursacht eine Zellnekrose, die jedoch nicht unmittelbar zu einer Größenabnahme des Zieltumors führt. In der ersten Bildgebung nach TACE, in der Regel ein bis drei Monate nach Therapie, ist daher von keiner Größenreduktion auszugehen. In einzelnen Fällen kann es hingegen aufgrund einer Ödementwicklung und Einblutungen zu einer geringen Größenzunahme kommen 8 . Eine Größenabnahme des behandelten Tumors wird jedoch im weiteren Verlauf beobachtet und führt im Langzeitverlauf zu einer Abnahme des Durchmessers.

Im Gegensatz zu der nur langsam eintretenden Größenabnahme des Zieltumors wird die Devaskularisation eines zuvor hypervaskularisierten Zieltumors unmittelbar nach TACE beobachtet und sollte im Verlauf der weiteren Nachsorge fortbestehen 30 . Während eine fokale, intratumorale Kontrastmittelaufnahme auf residuellen Tumor bzw. ein Lokalrezidiv hinweist, ist ein dünner, hyperarterialisierter Saum um den behandelten Tumor als physiologisch anzusehen ( Abb. 2 ) 31 . Zugrunde liegt eine Inflammation, die unmittelbar nach Therapie auftritt und über ein Jahr persistieren kann. Peripher gelegene, keilförmige Areale im Bereich des behandelten Tumors werden durch den embolisierenden Effekt der TACE verursacht und entsprechen dem behandelten Lebervolumen. Diese Veränderungen sind insbesondere in der arteriellen Phase als Minderkontrastierung ersichtlich, während es in späteren Phasen zu einem Angleichen der Kontrastierung mit dem umgebenden Gewebe kommt. Diese beschriebenen Perfusionsveränderungen lösen sich im längerfristigen Verlauf nach TACE auf 8 .

**Abb. 2:** HCC in Segment VIII bei einem 78-jährigen Patienten. **a** Planungsbildgebung mittels Cone-Beam-CT vor TACE. **b** Die erste MRT-Kontrollbildgebung 10 Wochen nach TACE zeigt ein dünnes randständiges Enhancement (Pfeil) des HCC in der spätarteriellen Phase der fettgesättigten T1-VIBE Sequenz, welches als therapieassoziiert zu werten ist.

Das Binnensignal eines mittels TACE behandelten Lebertumors stellt sich heterogen in der T1-Wichtung und vorwiegend hypointens in der T2-Wichtung dar. T2-hyperintense Areale können jedoch durch Einblutungen, liquide nekrotische Veränderungen oder ein reaktives Ödem verursacht werden, sodass eine Beurteilung des Therapieergebnisses anhand des Signalverhaltens erschwert wird 32 . T1-hyperintense Anteile können als Folge von Einblutungen oder proteinreichen Ablagerungen in dem behandelten Tumor auftreten. Um diese in der arteriellen Phase vom residuellen Tumor bzw. einem Rezidiv zu unterscheiden, können Subtraktionsbilder angefertigt werden ( Abb. 3 ).

**Abb. 3:** HCC in Segment V bei einer 69-jährigen Patientin. **a** Prätherapeutische Angiografie vor TACE eines HCC in Segment V (Pfeil). **b** In der ersten MRT-Kontrollbildgebung 4 Wochen nach Therapie stellt sich das behandelte HCC hyperintens (Pfeil) in der arteriellen Phase der fettgesättigten T1-VIBE-Sequenz dar. **c** Diese Hyperintensität ist jedoch bereits in der Nativphase abgrenzbar. **d** In den Subtraktionsbildern zeigt sich keine Kontrastmittelaufnahme. Die Hyperintensität ist auf Einblutungen oder proteinreiche Ablagerungen im Tumorresiduum zurückzuführen und darf nicht fälschlicherweise als vitaler Resttumor interpretiert werden.

Wie eingangs erwähnt, ist die MRT der CT bei der Beurteilung des Therapieeffektes nach TACE überlegen, da hier keine Beeinflussung durch Aufhärtungsartefakte durch das abgelagerte Lipiodol besteht. Vielmehr kann die Opposed-Phase T1-Sequenz auch verwendet werden, um eine Verteilung des Lipiodols innerhalb des Tumors zu beurteilen 8 .

## Thermische Ablation (RFA, MWA)

Das Ablationsareal lässt sich in der MRT-Bildgebung unmittelbar nach Therapie abgrenzen. Es stellt sich in aller Regel größer dar als der behandelte Tumor, bedingt durch den intendierten Sicherheitssaum von 5–10 mm und aufgrund der durch die Elektrode bzw. Antenne bedingten Mindestgröße der Therapiezone 33 . Die zuvor behandelte Läsion lässt sich in einigen Fällen unmittelbar nach Ablation innerhalb der Ablationszone noch eine Zeit lang abgrenzen ( Abb. 4 ). Das Binnensignal der Ablationszone kann sich heterogen darstellen. T1-hyperintense Areale resultieren häufig aus Einblutungen oder proteinreicher Nekrose, T2-Hyperintensität hingegen aus Ödem, Liquefaktion oder Gewebeumbau 34 .

**Abb. 4:** MRT-gesteuerte Mikrowellenablation. **a** Die Planungsbildgebung mittels fettgesättigter T1-VIBE zeigt das zu behandelnde HCC eines 72-jährigen Patienten in Segment VIII mit Hyperarterialisation (Pfeil). **b** Die Läsion zeigt in der Spätphase ein Washout. **c** Während der Intervention wird der Applikator bildgesteuert perkutan bis zur Läsion vorgeschoben (sagittale Schnittführung). **d** Die abschließende kontrastmittelverstärkte Kontrollbildgebung zeigt die ovalär konfigurierte Ablationszone, die sich scharf begrenzt minderkontrastiert gegenüber dem unbehandelten Leberparenchym darstellt (Pfeilkopf). Die avitale Läsion selbst ist noch residuell zentral in der Ablationszone abgrenzbar (Pfeil). **e** Punktionsbedingt kann es zur Ausbildung von arteriovenösen und arterioportalen Shunts kommen (anderer Patient), die sich in der arteriellen Phase peripher der Ablationszone in keilförmiger Konfiguration manifestieren. **f** In der Spätphase zeigen Shunts kein Washout.

**Verlauf nach MWA/RFA**

Randlich weist die Ablationszone typischerweise einen schmalen hyperperfundierten Randsaum auf, welcher auf eine postinterventionelle Hyperämie und reaktive Inflammation zurückzuführen ist und sich kontrastmittelaffin, jedoch ohne Washout darstellt. In diffusionsgewichteten Sequenzen weist dieser erniedrigte ADC-Werte auf 35 . Dieses Muster gilt als physiologisch und persistiert in Einzelfällen über mehrere Monate hinweg 4 8 . Im Gegensatz dazu ist das Auftreten von nodulären, unregelmäßigen Kontrastmittelanreicherungen innerhalb oder am Rand des Ablationsareals als suspekt für residuellen Tumor zu werten. Subtraktionsbilder aus arterieller und Nativphase können auch hier bei unklarer Hyperintensität helfen, um echtes arterielles Enhancement von Hämorrhagien differenzieren zu können 32 . Die DWI ist für die Beurteilung der zentralen Ablationszone nach Erfahrung der Autoren nur bedingt geeignet. Zwar sind Rezidive mit erniedrigten ADC-Werten und Nekrose mit erhöhten ADC-Werten beschrieben 36 , jedoch kann das Signal stark variieren.

Unmittelbar postinterventionelle, perihepatische Flüssigkeitsansammlungen können insbesondere bei weit peripher gelegenen Herden als Reaktion der Leberkapsel auf die thermische Schädigung auftreten und sind spontan regredient. Diese müssen jedoch unterschieden werden von relevanten Blutungen nach Retraktion der Elektrode bzw. des Applikators. Einzelne Luftfiguren im Bereich der Ablationszone sind typischerweise unmittelbar postinterventionell zu erkennen. Diese können bis zu zwei Wochen nach Therapie persistieren, sind als physiologisch zu werten und sollten nicht als Infektion interpretiert werden. Unmittelbar nach Ablation ersichtliche, keilförmige hypervaskularisierte Areale entsprechen arterio-venösen oder arterio-portalen Shunts, die durch die Punktion entstehen ( Abb. 4 **e** , **f** ). Diese Perfusionsveränderungen können über Wochen bis Monate persistieren. Im Gegensatz zu diesen vaskulären Veränderungen können im Verlauf von wenigen Wochen nach Ablation subsegmentale Gallengangserweiterungen peripher der Ablationszone auftreten. Diese Gallengangsveränderungen sind irreversibel, jedoch kein Zeichen einer rezidivbedingten Cholestase, sondern auf thermische Schädigung der Gallenwege zurückzuführen ( Abb. 5 ). Im Verlauf über Monate und Jahre hinweg schrumpft die Ablationszone und persistiert schließlich als narbiges signalarmes Residuum ( Abb. 6 ).

**Abb. 5:** Verlaufsbildgebung nach Mikrowellenablation. **a** In einer Verlaufskontrolle 6 Wochen nach Mikrowellenablation stellt sich die Ablationszone bereits größenregredient dar. Die kontrastmittelverstärkte T1-VIBE-Sequenz zeigt in der arteriellen Phase hyperintense Strukturen in der Ablationszone (Pfeil). **b** Die Hyperintensität lässt sich auch in der nativen Phase abgrenzen und ist durch Einblutungen und Proteinablagerungen erklärbar. **c** Die Subtraktion bestätigt, dass es sich hierbei nicht um vaskularisierte Anteile handelt. **d** Das Ablationsareal bleibt auch in der portalvenösen Phase minderkontrastiert gegenüber dem unbehandelten Leberparenchym. **e** In einer späteren Verlaufskontrolle 12 Wochen nach Therapie lassen sich dilatierte Gallenwege (Pfeilkopf) peripher der Ablationszone abgrenzen (T2-gewichtete Sequenz). Diese sind auf eine thermische Schädigung der Gallenwege zurückzuführen und nicht Ausdruck einer rezidivbedingten Cholestase. Diese Schädigungen sind irreversibel. **f** Coronare Schnittführung einer T2-gewichteten Sequenz. Die erweiterten Gallenwege sind hyperintens (Pfeilkopf). **g** In der kontrastmittelangehobenen T1-gewichteten Sequenz sind die Gallenwege hypointens.

**Abb. 6:** Langzeitverlauf nach perkutaner Mikrowellenablation eines HCC in Segment V. **a** Fettgesättigte T1-VIBE, arterielle Phase: fokales HCC mit Hyperarterialisation (Pfeil). **b** Spätphase: Washout der Läsion. **c** Erste Kontrolle 4 Wochen nach Ablation, arterielle Phase: ausgedehnte Ablationszone bis zur Leberkapsel; zentrale T1-Hyperintensitäten (Hämorrhagie/proteinreiche Nekrose); kein residuales arterielles Enhancement. **d** Erste Kontrolle 4 Wochen, Spätphase: minderkontrastierte Ablationszone mit schmalem, reaktiv hyperämischem Randsaum. **e** 10 Monate nach Ablation, arterielle Phase: deutliche Volumenregredienz der Ablationszone; zentrale Hyperintensitäten regredient. **f** 10 Monate, Spätphase: klare Abgrenzung des hypokontrastierten Areals; beginnende Kapselretraktion. **g** 28 Monate, arterielle Phase: weiterhin kein Nachweis vitaler Tumoranteile. **h** 28 Monate, Spätphase: weitere Schrumpfung der Ablationsnarbe und progrediente Kapselretraktion.

## SIRT (Selektive interne Radiotherapie)

Nach SIRT tritt das morphologische Therapieansprechen verzögert nach erst 3–6 Monaten ein 14 37 . Das arterielle Hyperenhancement mit oder ohne Washout des Tumors kann daher persistieren, ohne dass dies für ein Therapieversagen spricht ( Abb. 7 ) 38 . In den ersten Wochen nach Therapie zeigen sich häufig inhomogene Signalveränderungen in der MRT in T1- und T2-gewichteten Sequenzen. T1-Hyperintensitäten treten durch Blutabbauprodukte oder Proteinpräzipitate auf, T2-Hyperintensitäten im Rahmen von Nekrose oder Ödem. Initial bleibt das Tumorvolumen häufig stabil oder nimmt über die ersten vier Monate hinweg sogar leicht zu, bedingt durch inflammatorische Reaktionen oder intratumorale Einblutungen (Pseudoprogression) 8 . Ein randlicher schmaler Enhancementsaum (typischerweise <5mm) tritt in bis zu 50% der Fälle im Verlauf von einem halben Jahr auf und sollte aufgrund der zirkulären Konfiguration nicht als Tumorrezidiv missinterpretiert werden 32 39 . Noduläre arterielle Kontrastmittelanreicherungen können auch in erfolgreich behandelten Tumoren noch nach drei Monaten persistieren, sich im weiteren Verlauf jedoch regressiv darstellen 8 . Auch Granulationsgewebe kann sich potenziell in dieser Form darstellen und ist häufig nur über weitere Verlaufskontrollen zu unterscheiden. Gelegentlich treten strahleninduzierte Veränderungen im umliegenden Parenchym auf, erkennbar als geografisch-keilförmige Areale mit transienten Kontrastierungsveränderungen, die bis zu 6 Monate oder länger persistieren können und keinen infiltrativen Tumor darstellen. Fibrose mit möglicher Kapselretraktion und Volumenreduktion tritt nach Monaten in Erscheinung. Dieser Effekt ist besonders ausgeprägt bei der „Radiation Lobectomy“ zu beobachten, die neben der Atrophie des behandelten Leberlappens zu einer kompensatorischen Hypertrophie der unbehandelten Leber führt 40 .

**Abb. 7:** Verlauf nach SIRT eines HCC in Lebersegment III bei einem 53-jährigen Patienten (Z.n. Hemihepatektomie rechts). **a** Prätherapeutisch, fettgesättigte T1-VIBE, arterielle Phase: hypervaskularisiertes HCC nahe des Resektionsrandes (Pfeil). **b** Prätherapeutisch, Spätphase: Washout der Läsion. **c** 12 Wochen nach SIRT, arterielle Phase: dünnes randständiges Enhancement um die behandelte Läsion (Pfeilköpfe) im Sinne einer posttherapeutischen Hyperämie. **d** 12 Wochen, Subtraktion: persistierende zentrale Kontrastmittelaufnahme; nach SIRT kann die zentrale Arterialisation über Wochen bis Monate persistieren und darf nicht frühzeitig als Therapieversagen interpretiert werden. **e** 9 Monate nach SIRT, arterielle Phase: keine Hyperarterialisation mehr nachweisbar. **f** 9 Monate, Spätphase: nur diskretes Residuum abgrenzbar.

## Stereotaktische Strahlentherapie (SBRT)

Die morphologischen Veränderungen nach SBRT treten ebenfalls verzögert auf ( Abb. 8 , Abb. 9 ). In der Bildgebung bis etwa drei Monate nach Therapie bleibt die Tumorgröße meist unverändert oder kann geringfügig zunehmen 38 . In bis zu 75% der Fälle bleibt das arterielle Hyperenhancement über mehrere Monate bestehen, selbst bei histologisch bestätigter Nekrose, und bildet sich erst nach etwa 6–12 Monaten deutlich zurück 20 . Gelegentlich tritt ein dünnes randständiges Enhancement auf, welches weniger häufig und weniger ausgeprägt ist als bei der SIRT. Dieses Enhancement ist ebenfalls inflammatorisch bedingt und bildet sich meist im Verlauf von 6 Monaten zurück. Parallel dazu entwickeln sich ödematöse Veränderungen im peritumoralen Lebergewebe, oft als „focal liver reaction“ (FLR) bezeichnet und sichtbar als geografisch konfiguriertes T2-hyperintenses Areal mit arteriellem Hyperenhancement und möglichem Hypoenhancement in der portalvenösen Phase, welches bis zu einem Jahr oder sogar länger persistieren kann 20 38 . Vollständig nekrotische Tumoranteile weisen in diffusionsgewichteten Sequenzen erhöhte ADC-Werte auf (Navin et al., 2022). Nach etwa 6 Monaten kommt es zunehmend zur Ausbildung fibrotischer Veränderungen, die langfristig persistieren, und mit einer deutlichen Kapselretraktion einhergehen können.

**Abb. 8:** Verlauf nach SBRT eines subkapsulären HCC in Lebersegment VII. **a** Ein subkapsulär gelegenes HCC in Lebersegment VII (Pfeil) stellt sich prätherapeutisch hyperarterialisiert in der fettgesättigten T1-VIBE dar. **b** Die Läsion ist in der fettgesättigten T2-gewichteten Sequenz schwach hyperintens. **c** In einer Verlaufskontrolle sechs Wochen nach SBRT zeigt sich der Herd gering größenprogredient. **d** Peritumoral zeigen sich ödematöse Veränderungen im vormaligen Bestrahlungsfeld („focal liver reaction“). **e** In einer weiteren Kontrollbildgebung 12 Wochen nach SBRT ist die Arterialisation des Herdes deutlich regredient. **f** Ebenso sind die peritumoralen Signalveränderungen rückläufig. Die T2-hyperintensen Signalveränderungen im miterfassten rechten Unterlappen der Lunge sind durch eine Strahlenpneumonitis bedingt (Pfeilkopf).

**Abb. 9:** SBRT und TACE eines ausgedehnten HCC in Segment I bei einer 54-jährigen Patientin. **a** Prätherapeutisch, fettgesättigte T1-VIBE, arterielle Phase: ausgedehntes hypervaskularisiertes, histologisch gesichertes HCC in Segment I (Pfeil). **b** Prätherapeutisch, Spätphase: Washout der Läsion. **c** 6 Monate nach SBRT, arterielle Phase: keine Hyperarterialisation mehr nachweisbar; keine relevante Größenänderung der Läsion. **d** 6 Monate nach SBRT, Spätphase: Tumorgröße weiterhin unverändert. **e** 10 Monate nach SBRT, arterielle Phase: neue noduläre arterialisierte Herde entlang der posterioren Zirkumferenz der Läsion (Pfeilköpfe) als Zeichen eines Rezidivs; Empfehlung zur TACE in der interdisziplinären Tumorkonferenz. **f** 10 Monate, Spätphase: weiterhin keine relevante Größenänderung. **g** 7 Monate nach erfolgreicher TACE (insgesamt 17 Monate nach SBRT), arterielle Phase: kein Nachweis rezidivverdächtiger arterialisierter Läsionen. **h** 7 Monate nach erfolgreicher TACE, Spätphase: deutliche Größenregredienz der Läsion.

Der zeitliche Verlauf von therapieassoziierten Veränderungen nach Intervention ist in Abb. 10 schematisch dargestellt.

**Abb. 10:** Zeitlicher Verlauf therapieassoziierter Veränderungen nach interventioneller Therapie.

## Nachsorgeempfehlungen und Follow-Up Protokolle

Empfehlungen zur bildgebenden Nachsorge nach lokoregionärer Therapie des HCC sind sowohl in der deutschen S3-Leitlinie als auch in der europäischen EASL Clinical Practice Guideline festgehalten. Beide Leitlinien bevorzugen den Einsatz einer multiphasischen dynamischen MRT, wobei die kontrastverstärkte CT-Untersuchung eine valide Alternative darstellt 3 41 .

Nach Thermoablation, SIRT und TACE sollte laut S3-Leitlinie die initiale bildgebende Kontrolle zwischen 4 und 12 Wochen erfolgen, während für die SBRT eine erste Kontrolle frühestens nach 12 Wochen empfohlen wird. Ziel dieser ersten Kontrollen ist die Beurteilung des technischen Erfolgs sowie das Erkennen von Resttumorgewebe.

Im weiteren Verlauf des ersten Jahres empfiehlt die S3-Leitlinie regelmäßige bildgebende Kontrollen in einem Intervall von etwa 3 Monaten. Dieses engmaschige Vorgehen ist durch die deutlich erhöhte Rezidivwahrscheinlichkeit innerhalb des ersten Jahres begründet, welche etwa 6,5-fach höher ist als in späteren Zeiträumen 42 .

Im zweiten Jahr kann das Kontrollintervall auf 3 bis 6 Monate verlängert werden. Die S3-Leitlinie empfiehlt eine Nachsorgedauer von mindestens zwei Jahren, wobei die Fortführung individuell nach Rezidivrisiko und Patientenprofil auch länger erfolgen kann. Hausintern an der Institution der Autoren ist eine Ausdehnung der Nachsorge auf fünf Jahre üblich.

Nach Abschluss der Nachsorgephase sollen Patienten gemäß beiden Leitlinien in die reguläre Ultraschall-Früherkennung mit halbjährlichen Kontrollen überführt werden. Diese Empfehlung gilt insbesondere bei Patienten mit bestehender Leberzirrhose oder erhöhtem Rezidivrisiko aufgrund einer chronischen Lebererkrankung.

## Zusammenfassung

Die Bildgebung spielt eine zentrale Rolle in der postinterventionellen Beurteilung des Therapieansprechens beim hepatozellulären Karzinom. Art und zeitliche Dynamik der bildmorphologischen Veränderungen variieren in Abhängigkeit von der eingesetzten Therapieform. Nach Thermoablation zeigt sich unmittelbar eine Ablationszone, die im Verlauf schrumpft. Nach erfolgreicher TACE tritt unmittelbar ein Rückgang des arteriellen Hyperenhancements auf, die Auswirkungen der zytotoxischen Effekte stellen sich erst nach Wochen ein. Nach SIRT und SBRT treten morphologische Veränderungen hingegen verzögert auf. Initiale Größenstabilität darf dabei nicht mit fehlendem Therapieansprechen verwechselt werden. Zentrale Elemente der radiologischen Beurteilung sind arterielle Kontrastmittelaufnahme und Washout. Die Anfertigung von Subtraktionsbildern kann dabei hilfreich sein. Die Kenntnis typischer Fallstricke – etwa postinterventionell inflammatorisch bedingte Hyperämie – ist essenziell, um Fehlinterpretationen zu vermeiden.

Die Bildgebung sollte sich an standardisierten Bewertungssystemen wie mRECIST oder LI-RADS-TR orientieren, welche den vitalen Tumoranteil interpretieren. Eine reine Betrachtung morphologischer Größenveränderungen wie etwa im Rahmen von RECIST 1.1 spiegelt den Therapieerfolg nicht ausreichend wider. Die aktualisierte LI-RADS-TR-Version 2024 berücksichtigt mit der Kategorie „non-progressing“ erstmals strahlenassoziierte Veränderungen und vereinfacht die Definition der Tumorvitalität. Ancillary features wie Diffusionsrestriktion oder T2-Hyperintensität ermöglichen zudem eine differenziertere Einordnung unklarer Befunde und tragen zu einer standardisierten Bewertung bei.

Limitationen dieser Übersichtsarbeit ergeben sich aus der Heterogenität der postinterventionellen Befunde: Nicht alle beschriebenen Veränderungen treten in jedem Fall auf, ihre zeitliche Ausprägung kann stark variieren und Kombinationstherapien können das Bild zusätzlich beeinflussen. Die Auswirkung von Systemtherapien wurde in der vorliegenden Arbeit nicht berücksichtigt, wodurch die Übertragbarkeit auf multimodale Therapiekonzepte eingeschränkt ist.
